# Growth differentiation factor 15 is a promising diagnostic and prognostic biomarker in colorectal cancer

**DOI:** 10.1111/jcmm.12830

**Published:** 2016-03-15

**Authors:** Chen Li, Xiaobing Wang, Ignacio Casal, Jingyu Wang, Peiwei Li, Wei Zhang, Enping Xu, Maode Lai, Honghe Zhang

**Affiliations:** ^1^ Department of PathologySchool of MedicineZhejiang UniversityHangzhouChina; ^2^ Key Laboratory of Disease Proteomics of Zhejiang ProvinceHangzhouChina; ^3^ Medical Center for Tumor DetectionCancer Institute and HospitalChinese Academy of Medical Sciences and Peking Union Medical CollegeBeijingChina; ^4^ Department of Cellular and Molecular MedicineCentro de Investigaciones Biológicas (CIB‐CSIC)MadridSpain; ^5^ Department of Pathologythe First Hospital of JiaxingJiaxingChina; ^6^ Department of Epidemiology and Health StatisticsZhejiang University School of Public HealthHangzhouChina

**Keywords:** colorectal cancer, growth differentiation factor 15, diagnostic, prognostic, meta‐analysis

## Abstract

Although various studies have demonstrated that growth differentiation factor 15 (GDF15) might be a potential diagnostic and prognostic marker in colorectal cancer (CRC) patients, the results are inconsistent and the statistical power of individual studies is also insufficient. An original study was conducted to explore the diagnostic and prognostic value of serum GDF15 in CRC patients. We also conducted a meta‐analysis study which aimed to summarize the diagnostic and prognostic performance of serum GDF15 in CRC. We searched PubMed and ISI Web of Knowledge up to 1 November 2014 for eligible studies. In order to explore the diagnostic performance of GDF15, standardized mean difference (SMD) and their 95% confidence intervals (CI) were estimated and receiver‐operating characteristic (ROC) curves were constructed. For prognostic meta‐analysis, study‐specific hazard ratios (HRs) of serum GDF15 for survival were summarized. A total of eight studies were included in the meta‐analyses. Our results revealed that serum GDF15 levels in CRC patients were higher than those in healthy controls (SMD = 1.08, 95% CI: 0.56–1.59, *P* < 0.001). For discriminating CRC from healthy controls, the AUC of GDF15 was 0.816 (95% CI: 0.792–0.838). The sensitivity and specificity were 58.9% (95% CI: 55.0–62.8) and 92.08% (95% CI: 89.2–94.4), respectively, when a cut‐off value of 1099 pg/ml was established. Besides, higher GDF15 expression level was associated with worse overall survival for CRC patients (pooled HR = 2.09, 95% CI: 1.47–2.96). In conclusion, the present meta‐analysis suggests that serum GDF15 may be a useful diagnostic and prognostic biomarker for CRC.

## Introduction

Colorectal cancer (CRC) remains a major cause of cancer mortality worldwide. Early detection and surgical excision of pre‐cancerous polyps could minimize morbidity and mortality from CRC. As suggested, CRC screening strategies have reduced 44% risk of CRC, compared with patients who never undergo screening 1. CRC screening modalities include colonoscopy, faecal occult blood test, stool DNA tests, barium enema and colon video capsule, and colonoscopy remains the gold standard for the early detection of adenoma or CRC 2. However, those screening methods are still inadequate because of their low sensitivity or specificity, high invasiveness or high cost. Novel technologies, especially the identification of new biomarkers, are necessary for improving CRC early diagnosis.

Growth differentiation factor 15 (GDF15) is a divergent member of the transforming growth factor‐β (TGF‐β) superfamily 3. GDF15 is also known as macrophage inhibitory cytokine‐1 (MIC‐1), prostate‐derived factor (PDF), placental TGF‐β (PTGF‐β), placental bone morphogenetic protein and non‐steroidal anti‐inflammatory drug‐activated gene‐1 (NAG‐1) 4, 5, 6, 7, 8, 9. GDF15 expression level is usually low in resting cells but may be substantially increased following response to diverse cellular stress signals, such as hypoxia, inflammation, short‐wavelength light exposure, acute tissue injury and during cancer progression 10, 11, 12. The deregulation of GDF15 expression has been associated with diverse human disease development and cancer progression 13, 14, 15, 16, 17, 18, 19, 20. GDF15 level was increased in the serum of patients with various cancers, including melanoma, oral squamous cell, gastrointestinal, colorectal, pancreatic, prostate, breast and cervical epithelial 13, 16, 18, 21, 22, 23. Moreover, GDF15 is also overexpressed in adenomatous colonic polyps 24. Of clinical interest, GDF15 levels were associated with an increased risk of recurrent adenoma 25, and higher GDF15 levels in serum have been correlated with poor prognosis in cancer patients 26, 27, 28, 29.

Many studies have evaluated the application of GDF15 in the diagnosis and prognosis of CRC; however, there are some discrepant results which have been reported, and the statistical power of individual studies may be insufficient. Therefore, we performed this meta‐analysis of all relevant available data to explore the diagnosis and prognosis performance of serum GDF15 in CRC.

## Materials and methods

### Original study and associated statistical analysis

We conducted an original study to explore the diagnostic and prognostic value of GDF15 expression in CRC. Serum samples of 138 CRC patients were collected from the Sir Run Run Shaw Hospital, Zhejiang University, between 2008 and 2010. The control group consisted of 171 healthy blood donors from routine healthy examinations during the same period. The study was approved by the Ethics Board of Biomedicine, Zhejiang University, China. Patients who received R0 resection were included and who had received pre‐operative chemotherapy or radiotherapy was excluded from the study. Informed consent was obtained from all the included patients. The plasma samples were aliquoted into RNase‐free tubes after centrifugation at 4°C. The serum was stored at −80°C until use. Samples were thawed before analyses. Serum levels of GDF15 were measured using sandwich enzyme‐linked immunosorbent assays (ELISA; catalogue number MAB957; R&D Systems, Minneapolis, MN, USA).

The level of GDF15 in serum was presented as mean ± standard deviation (S.D.). GDF15 expression levels were compared between CRC patients and controls using a non‐parametric test. Survival curves were conducted using the Kaplan–Meier method. Cox's proportional hazard regression analyses were performed to calculate hazard ratios (HRs) of death according to GDF15 expression levels. Multivariate models were applied to adjust potential influence factors for death, including age, sex and TNM stage. The statistical analyses were conducted with SPSS 20.0 for Windows (IBM Corp., Armonk, NY, USA). *P* values <0.05 were considered statistically significant.

### Meta‐analysis study

The systematic review and meta‐analysis study was performed in accordance with the Preferred Reporting Items for Systematic reviews and Meta‐Analyses (PRISMA) guidelines 30.

#### Literature search, study selection, data extraction, quality assessment

Study selection, data extraction, quality assessment, and data synthesis were carried out by two independent reviewers. Any discrepancies were resolved through discussion or by a third reviewer. Literature search was performed in PubMed and ISI Web of Knowledge up to November 2014. The following keywords were used: (‘colorectal’ OR ‘colon’ OR ‘rectal’) AND (‘cancer’ OR ‘tumor’ OR ‘carcinoma’ OR ‘adenocarcinoma’) AND (‘GDF15′ OR ‘GDF‐15′ OR ‘growth differentiation factor 15′ OR ‘MIC1′ OR ‘MIC‐1′ OR ‘macrophage inhibitory cytokine‐1′ OR ‘NAG1′ OR ‘NAG‐1′ OR ‘non‐steroidal anti‐inflammatory drug‐activated gene‐1′ OR ‘PDF’ OR ‘prostate‐derived factor’ OR ‘PTGF‐β’ OR ‘placental TGF‐β’ OR ‘PLAB’ OR ‘placental bone morphogenetic protein’). Reference lists of the included articles or relevant reviews were also browsed for potentially missing studies. The retrieved studies were screened to exclude potential duplicates or overlapping data. For study selection, initial screening by scanning titles and abstracts of articles were conducted, and then full text of potential eligible studies was assessed.

Articles were included if they met all the following criteria: (1) the study evaluated diagnostic or prognostic value of blood GDF15 level in CRC patients; (2) for studies that analysed the diagnostic value of GDF15, sensitivity and specificity were reported or could be calculated; and (3) for prognostic studies, HR values with 95% confidence interval (CI) were provided or could be calculated. Characteristics of the included studies and data of GDF15 expression were extracted and listed as follows: first author, year of publication, origin of the study population, patient characteristics (age, sex, cancer type and stage), type of biological specimen, number of participants, GDF15 assay method, follow‐up time and variables adjusted for in the analysis. For diagnostic studies, the expression of GDF15 levels or sensitivity and specificity, or relative risk, or odds ratio (OR) estimates, and their 95% CI were extracted. For prognostic studies, HR with 95% CIs was extracted. The corresponding author or the first author was contacted if the information of the publication was incomplete.

If the study provided medians and interquartile ranges instead of mean and S.D., we computed the mean and S.D. as described by Hozo *et al*. 31. We calculated the lower and upper ends of the range by multiplying the difference between the median and upper and lower ends of the interquartile range by 2 and adding or subtracting the product from the median, respectively 32. The Quality Assessment of Studies of Diagnostic Accuracy included in Systematic Review (QUADAS) 33 assessment tool, which contains 14 items, was applied for the quality assessment of diagnostic studies of GDF15 (Table S1). REMARK tool 34 was used to evaluate prognostic studies of GDF15.

#### Statistical analysis

Receiver‐operating characteristic (ROC) analysis was performed, and the area under the curve (AUC) was assessed to determine the diagnostic performance of serum GDF15 level in CRC. Sensitivity and specificity with their 95% CIs were calculated for the best cut‐off value. The ROC curves were calculated and compared with MedCalc version 12.4.

The difference of GDF15 between CRC and controls was estimated by standard mean difference (SMD) and its 95% CI. Study‐specific HR estimates were pooled for prognostic evaluation of GDF15 in CRC. The extent of heterogeneity across studies was quantified by inconsistency index (*I* ^2^) and confirmed significant with *P* value, and *P* ≤ 0.10 and/or *I* ^2^ > 50% indicates significant heterogeneity 35. Diagnostic and prognostic parameters were pooled using the fixed‐effects model if there was no significant heterogeneity; otherwise, a random‐effects model was adopted. Funnel plot was applied to check whether there was obvious publication bias among the involved studies 36.

The meta‐analysis was performed using STATA software (Stata Corporation, College Station, TX, USA, version 12.0 for windows). *P* < 0.05 was considered statistically significant.

## Results

### The original study

**Figure 1:**
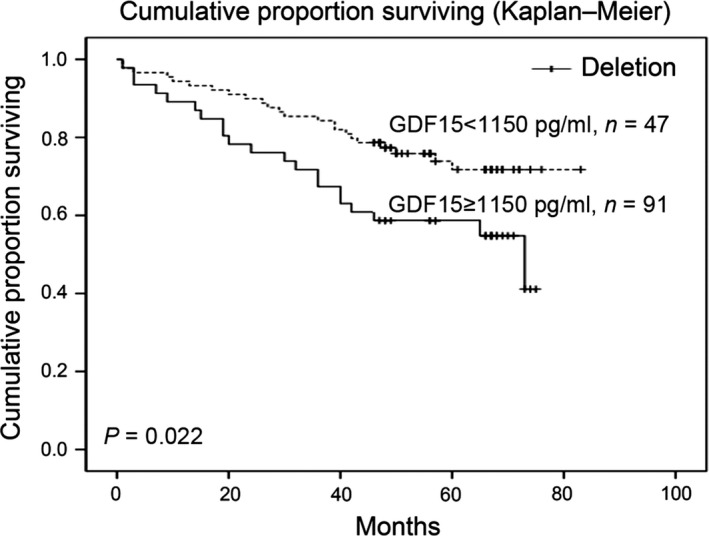
Kaplan–Meier curve of GDF15 expression in relation to overall survival of CRC patients.

A total of 138 CRC patients and 171 healthy controls were included to assess the diagnostic and prognosis value of serum GDF15. We found that the serum level of GDF15 was significantly higher in CRC patients than in controls (745.78 ± 308.79 pg/ml in controls *versus* 1075.31 ± 481.35 pg/ml in CRC patients, *P* < 0.001). This suggested that serum GDF15 was a promising marker in discriminating CRC from controls. According to Brown *et al*. 27 and Wallin *et al*. 26 reports, we choose 1150 pg/ml to differentiate GDF15 serum levels. Patients with higher serum levels of GDF15 had a shorter overall survival (*P* = 0.022) (Fig 1). As the multivariate Cox proportional hazard regression analysis indicated, GDF15 appeared to be an independent prognostic marker for CRC patients (HR = 1.915; 95% CI: 1.014–3.617; *P* = 0.045).

### The meta‐analysis

#### Study selection and characteristics

**Figure 2:**
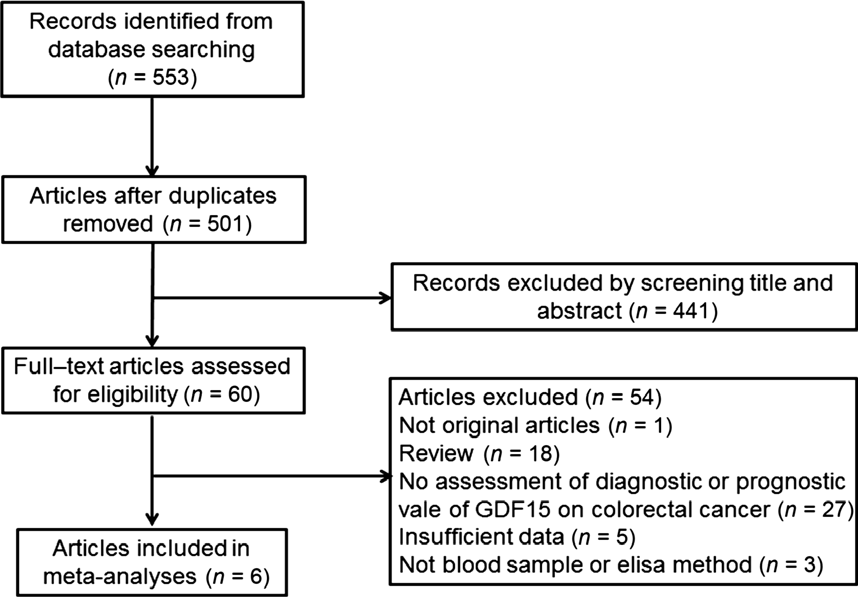
Flow diagram of the study selection process.

The process of study selection is shown in Figure 2. After screening titles and/or abstracts, irrelevant studies were excluded and 60 studies were evaluated through detailed reading. Among them, 54 were excluded for the following reasons: we could not obtain full text for one study, 18 publications were reviews, 27 studies did not evaluate the diagnostic or prognostic value of GDF15 in CRC, data from five studies were incomplete and three studies use other methods, like RT‐PCR or IHC to detect GDF15 levels. Six studies met our eligibility criteria 26, 27, 37, 38, 39, 40. With our original study observations and unpublished data from Wang *et al*., eight studies were finally included in the meta‐analyses. The characteristics of the included studies were presented in Table S2. Among the included studies, seven articles reported the diagnostic value of GDF15 (including our original study and unpublished data from Wang *et al*.; besides, study conducted by Mehta *et al*. including two cohorts, the NHS and the HPFS), while three studies examined the prognostic value of GDF15 (including one original study) and two studies involved both prognostic and diagnostic value.

### Diagnostic value of circulating GDF15 for CRC

The difference of GDF15 levels in CRC and healthy controls was reported in a combined seven studies with 3709 participants. The included studies were conducted in China (*n* = 4), the United States (*n* = 1), Australia (*n* = 1) and Spain (*n* = 1). Sample size of each study ranged from 60 to 805. The expression of GDF15 in serum was determined by ELISA. The quality assessments were showed in Table S3.

**Figure 3:**
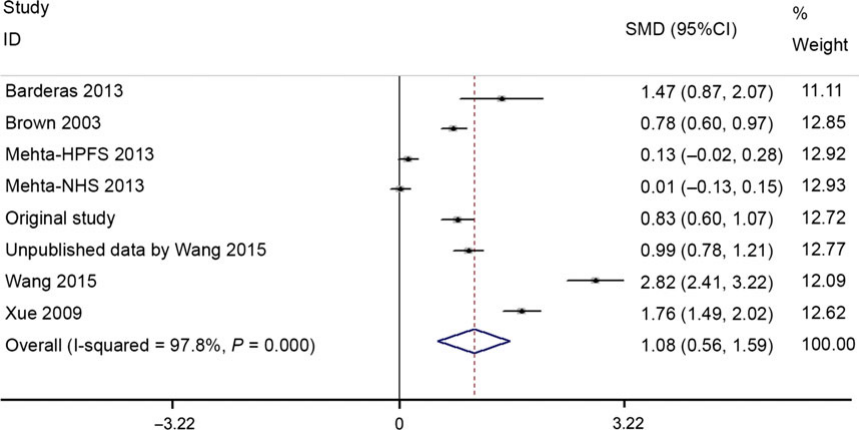
Forest plots of SMD of GDF15 between CRC patients and healthy controls.

GDF15 levels in CRC patients ranged from 754 ± 519.3 to 2216.5 ± 1496.9 pg/ml, whereas levels in the healthy control group ranged from 406.9 ± 239 to 910.7 ± 293.2 pg/ml. The random‐effects model was conducted due to the significant heterogeneity across studies. The meta‐analysis results revealed that GDF15 levels were significantly lower in healthy controls compared with CRC patients (pooled SMD = 1.08, 95% CI: 0.56–1.59; *P* < 0.001), and significant heterogeneity was found: I^2^ = 97.8% (Fig. 3).

**Figure 4:**
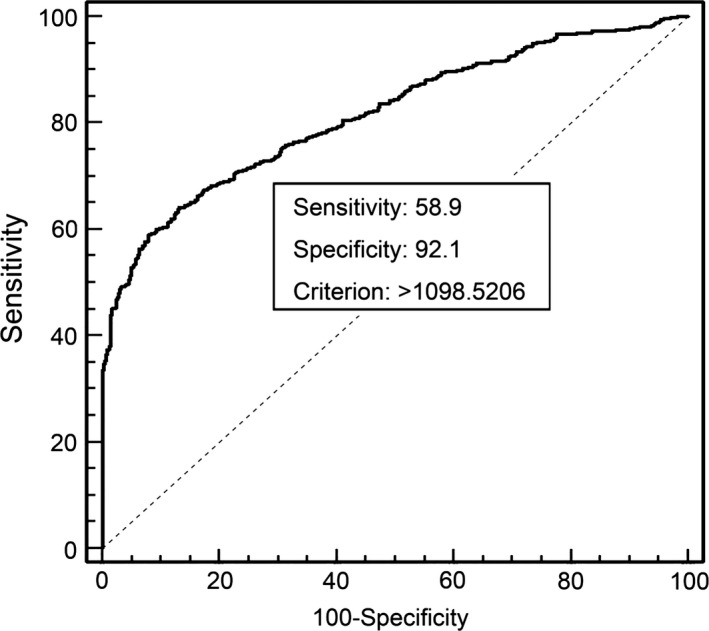
ROC curve analysis for the diagnostic accuracy of serum GDF15 levels.

A total of four studies with 1104 participants evaluated the diagnostic accuracy of GDF15. The ROC analyses yielded an AUC of 0.816 (95% CI: 0.792–0.838). At a cut‐off value of 1099 pg/ml, the sensitivity and specificity of GDF15 was 58.9% (95% CI: 55.0–62.8) and 92.08% (95% CI: 89.2–94.4) separately for differentiating CRC from healthy controls. The ROC curves of serum GDF15 for predicting CRC is shown in Figure 4.

### Prognosis value of GDF15 for CRC

There were three studies (including our present study) with 422 patients that evaluated the influence of GDF15 expression on CRC overall survival. The included studies were conducted in China (*n* = 1), Australia (*n* = 1) and Sweden (*n* = 1). The quality assessments are shown in Table S4.

**Figure 5:**
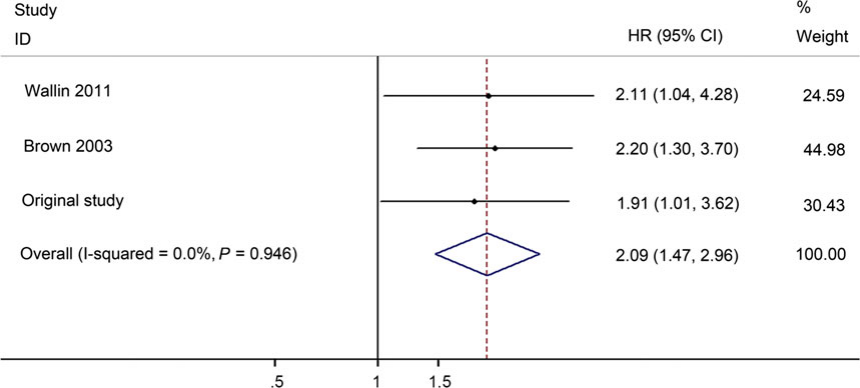
Forest plots of overall survival of GDF15 in the prognosis of CRC.

The pooled HR was 2.09 (95% CI: 1.47–2.96), indicating that higher GDF15 expression level was associated with poorer overall survival for CRC patients (Fig. 5).

### Publication bias and sensitivity analysis

Begg's funnel plot and Eegg's linear regression test were performed to estimate the potential publication bias of the included studies. No obvious asymmetry was found in the funnel plots of the diagnostic and prognostic meta‐analyses.

Sensitivity analysis was conducted by omitting one study at a time and recalculated the pooled SMD and HR for the remaining studies. The results suggested that no individual studies substantially influenced the pooled point estimate.

## Discussion

Colorectal cancer is one of the leading causes of cancer‐related deaths worldwide. CRC is thought to develop from malignant degeneration of adenomatous polyps *via* a well‐established gradual progression from adenoma to carcinoma 41. However, survival of patients largely depends on the stage of CRC at diagnosis. The 5‐year survival is 90% when CRC is diagnosed at an early stage 42. Clinical trials have indicated that screening using faecal occult blood testing or other methods increases the detection rate of early‐stage disease and reduces the mortality of CRC 43. Besides, the efficacy of endoscopic polypectomy in preventing adenomas from progressing to CRC has led to a decrease in the incidence of CRC 44. Although significant advances has been achieved in the diagnosis and prognosis of CRC, exploration of better biomarkers is still important for CRC early detection and for predicting patients’ outcome.

Many studies have revealed the important role of GDF15 in CRC. It has been observed that GDF15 expression levels are markedly enhanced in malignant tissues, established cancer cells and plasma during the transition of numerous localized cancers to invasive and metastatic disease stages as compared with non‐malignant tissues, normal cells, and basal GDF15 concentration in serum 12, 13, 14, 15, 16, 17, 18, 19, 20, 26, 27, 28, 29, 38, 39, 40, 41. GDF15 deregulation is involved in the progression of colon cancer, and research showed that the GDF15 levels in serum gradually increase in the process of conversion from adenomatous polyps to colorectal carcinoma 27. There is a significant positive relationship between serum GDF15 levels, clinical stage, presence of metastasis and progression of CRC 27. Immunohistochemical analysis of GDF15 expression in CRC has also been associated with lymph node metastasis 38.

These suggest that the assessment of GDF15 level may have clinical use. Currently, some reports explored the relationship between GDF15 serum levels and some biomarkers in cancers. Carcinoembryonic antigen (CEA) has been extensively used as biomarker for recurrence and metastasis in CRC, and there was a correlation between the GDF15 serum levels and CEA 26, 27. In prostate cancer, CA19‐9 widely used as diagnostic marker 45, 46. The diagnosis efficiency of GDF15 could be comparable to CA19‐9 in prostate cancer 18. GDF15 level and cancer antigen 19‐9 (CA 19‐9) significantly improved diagnostic accuracy of the patients with pancreatic ductal adenocarcinoma 15, 18. The expression of GDF15 and prostate‐specific antigen has been reported to significantly improve the diagnostic specificity 47. In addition, the detection of GDF15 has some practical advantages. There were no significant differences in GDF15 levels between various components of blood, and samples need no special treatment 48. In serial studies, the level of GDF15 was relatively stable, comparable and reliable 18, 27, 49, 50. The means of GDF15 detection in serum was feasible, convenient and low cost. Even so, there were several deficiencies in standard and quality assurance among different studies. Well‐designed prospective studies and larger‐scale measurements of GDF15 are required to evaluate the value of GDF15 in CRC.

As the present meta‐analysis suggested, serum GDF15 level of CRC patients was higher than that in healthy controls (SMD = 1.08, 95% CI: 0.56–1.59). Further analyses revealed that GDF15 achieved a sensitivity of 58.9%, specificity of 92.08% and AUC of 0.816, indicating that GDF15 has a good diagnostic performance for CRC. Moreover, we demonstrated that GDF15 expression level could be a prognostic biomarker in CRC patients. Compared with patients with low GDF15 expression level, patients with an increased level of GDF15 had a 2.09‐fold higher risk of death. These results suggested that GDF15 could serve as a diagnostic and prognostic biomarker in CRC.

This systematic review had several advantages. First, we conducted a thorough literature search and comprehensively evaluate the diagnostic and prognostic value of GDF15 in CRC. Second, we obtained original data to explore the diagnostic accuracy and prognostic potential of GDF15 in CRC. In addition, the methods of this study were rigorous and followed the guidelines for conducting and reporting systematic reviews.

However, our study also has several limitations. First, the methodological quality of included studies was uneven. Diagnostic accuracy studies required ELISA standardization, which defines the normal range and objective threshold for discriminating positive and negative results in clinical studies. Second, only seven studies were included in the meta‐analyses, and the available information was insufficient for subgroup analyses; therefore, it was difficult to draw a definitive conclusion for its ability to discriminate. Moreover, significant heterogeneity was observed in the diagnostic meta‐analysis, and sample size and heterogeneous population could not fully explain the observed heterogeneity. Third, converting non‐normally distributed statistics (median and range) to normally distributed statistics (mean and S.D.) may have caused bias in our analysis 31, 32. Additionally, we only included English‐language articles, and thus language bias may have influenced the results.

## Conclusions

The current analysis showed that GDF15 is probably adequate to discriminate and distinguish CRC patients from healthy controls. Besides, higher GDF15 level was associated with poorer overall survival for CRC patients. In conclusion, this meta‐analysis suggests that GDF15 is a promising biomarker with diagnostic and prognostic value for CRC. Well‐designed prospective studies with large patient cohorts are required to reliably evaluate the value of GDF15 as a biomarker in CRC.

## Author contributions

H. Zhang and M. Lai conceived the project and supervised research. C. Li and H. Zhang prepared the manuscript. X. Wang, I. Casal. and W. Zhang provided the original data. J. Wang designed and performed the experiments. P. Li collected the clinical samples. E. Xu helped to perform statistical analysis. All authors reviewed the manuscript.

## Conflict of interest

The authors declare no conflict of interest.

## Supporting information


**Table S1** Instrument used for the evaluation of the risk of bias and applicability concerns of the included prognostic studies (adapted from QUADAS‐2).
**Table S2** Characteristics of studies included in the diagnostic meta‐analysis.
**Table S3** Quality assessment of the included diagnostic studies according to QUADAS‐2.
**Table S4** Definitions of 17 items of study reporting quality.
