# Supplementary material for: Growth differentiation factor 15 is a promising diagnostic and prognostic biomarker in colorectal cancer
Source: J Cell Mol Med. 2016 Mar 15;20(8):1420–6. doi: 10.1111/jcmm.12830 (PMC4956952; doi:10.1111/jcmm.12830)
Supplement: Supplementary file 1 — Table S1 Instrument used for the evaluation of the risk of bias and applicability concerns of the included prognostic studies (adapted from QUADAS‐2). Table S2 Characteristics of studies included in the diagnostic meta‐analysis. Table S3 Quality assessment of the included diagnostic studies according to QUADAS‐2. Table S4 Definitions of 17 items of study reporting quality. [file JCMM-20-1420-s001.docx]

**Supplementary Table 1** Instrument used for the evaluation of the risk of bias and applicability concerns of the included prognostic studies (adapted from QUADAS-2)

| **Domain** | **Patient Selection** | **Prognostic markers** | **Outcome** | **Flow** | **Timing** | **Confounding** |
| --- | --- | --- | --- | --- | --- | --- |
| Description | Describe methods of  patient selection  Describe included  patients (previous  testing, presentation,  intended use of index  test, and setting) | Describe the  prognostic marker and  how it was conducted  and interpreted | Describe the outcome  measurement and how  it was conducted and  interpreted | Describe any patients  who did not had the  prognostic markers  measured or for whom  outcome was not  measured or who were  excluded from the 2 X  2 table (refer to flow  diagram) | Describe the interval  and any interventions  between prognostic  markers and the  outcome  measurement | Describe any method  used to control for  potential confounding |
| Signaling  questions (yes,  no, or unclear) | Was a consecutive or  random sample of  patients enrolled?  Was a case–control  design avoided?  Did the study avoid  inappropriate  exclusions? | Were the prognostic  marker results  interpreted without  knowledge of clinical  data? (blinding)  If a threshold was  used, was it  prespecified? | Was the outcome  measurement  adequate?  Did all patients had  their outcome  evaluated the same  way?  Were the outcome  results interpreted  without knowledge of  the results of the  prognostic markers?  (blinding) | Did all patients have  outcome measured?  Were all patients  included in the  analysis?  (lost to follow-up?  withdrawal? patients  not tested? missing  data? etc.) | Was there an  appropriate interval  between prognostic  marker and outcome  measurement?  (sufficient time for  outcome to occur?) | Did the study  controlled for  potential  confounders? |
| Risk of bias (high,  low, or unclear) | Could the selection of  patients have  introduced bias? | Could the conduct or  interpretation of the  prognostic marker  have introduced bias? | Could the outcome  measurement, its  conduct, or its  interpretation have  introduced bias? | Could the patient flow  have introduced bias? | Could the timing have  introduced bias? | Confusion bias? |
| Concerns about  applicability (high,  low, or unclear) | Are there concerns  that the included  patients do not match  the review question? | Are there concerns  that the prognostic  marker, its conduct, or  its interpretation differ  from the review  question? | Are there concerns  that the outcome  measure does not  match the review  question?  (not clearly reported  according to prognostic  marker results or  different  dichotomisation of  scales, etc.) |  |  |  |

**Supplementary Tabl**e 2 Characteristics of studies included in the diagnostic meta-analysis

| Study | Origin of population | Cancer type | TNM Stage (n) | Age  (years) | Sex(M /F) | Source of samples | No. of participants | No. of cases/ controls | Endpoints  (Results) | Median/Mean follow- up time  (Years) | Adjusted factors | GDF15 assay |
| --- | --- | --- | --- | --- | --- | --- | --- | --- | --- | --- | --- | --- |
| Barderas /2013 | Spain | CRC | I (6)  II (7)  III (12)  IV (15) | Cases: 67.5  Controls: 60.5 | 35/25 | Serum | 60 | 40/20 | N/A | N/A | N/A | ELISA |
| Brown /2003 | Australia | CRC | I-IV | Cases: 66.6  Controls: 48 | 280/208 | Serum | 488 | 227/261 | N/A | N/A | N/A | ELISA |
| Mehta-NHS/2013 | USA | CRC | Nr | 58.9 | 0/763 | Serum | 763 | 344/419 | N/A | N/A | N/A | ELISA |
| Mehta-HPFS/2013 | USA | CRC | Nr | 65.8 | 805/0 | Serum | 805 | 274/531 | N/A | N/A | N/A | ELISA |
| Wang/2014 | China | CRC | I (5)  II (14)  III (7)  IV (4) | Nr | 305/225 | Serum | 530 | 30/500 | N/A | N/A | N/A | ELISA |
| Xue/2009 | China | CRC | I/II (68)  III/IV(76) | Cases:59  Controls: 56 | 162/138 | Serum | 300 | 144/156 | N/A | N/A | N/A | ELISA |
| Unpublished data by Wang/2015 | China | CRC | I (33)  II (103)  III (127)  IV (67) | Cases: 58.5  Controls: 57.5 | 255/199 | Serum | 454 | 330/124 | N/A | N/A | N/A | ELISA |
| Original study | China | CRC | I (16)  II (52)  III (30)  IV (40) | 59 | 177/132 | Serum | 309 | 138/171 | N/A | N/A | N/A | ELISA |
| Brown /2003 | Australia | CRC | I-IV | 66.6 | 141/86 | Serum | 227 | N/A | OS | 5 | Gender, Lymph nodes present and TNM stage | ELISA |
| Wallin/2011 | Sweden | CRC | I-IV | Nr | Nr | Serum | 57 | N/A | OS | 6 | Gender, hereditary for CRC, N-substage and neural  invasion | SP-PLA |
| Original study | China | CRC | I (16)  II (52)  III (30)  IV (40) | 59 | 79/59 | Serum | 138 | N/A | OS | 6 | Age, gender and TNM stage | ELISA |

Nr: not reported; N/A: not applicable; CRC: colorectal cancer; ELISA: enzyme-linked immunosorbent assay; OS: overall survival.

**Supplementary Table 3** Quality assessment of the included diagnostic studies according to QUADAS-2

| **Study** | **Risk of bias** | | | | **Applicability concerns** | | |
| --- | --- | --- | --- | --- | --- | --- | --- |
|  | Patient Selection | Index Test | Reference Standard | Flow and Timing | Patient Selection | Index Test | Reference Standard |
| Barderas /2013 | Unclear | Unclear | Unclear | Unclear | Low | Low | Low |
| Brown /2003 | Unclear | Unclear | Low | Unclear | Low | Low | Low |
| Mehta/2013 | High | High | Low | Unclear | Low | Low | Low |
| Wang/2014 | High | Low | Low | Low | Low | Low | Low |
| Xue/2009 | High | Low | Low | Unclear | Low | Low | Low |
| Original study | High | Low | Low | Low | Low | Low | Low |
| Unpublished study by Wang/2015 | High | Low | Low | Low | Low | Low | Low |

| **Supplementary Table 4. Definitions of 17 items of study reporting quality** |
| --- |
| Study Design   1. Objectives or prespecified hypothesis   State the study objectives, protocol or pre-specified hypothesis   1. Sample size   State a statistical sample size or power calculation   1. Follow-up description   State the follow-up period or the median follow-up time   1. Patients source   State health care setting from which patients were recruited   1. Patients selection criteria   State inclusion or exclusion criteria   1. Population characteristics   State the patients characteristics (e.g. age, gender and tumor stage)   1. Flow of patients   State the number of patients included in each stage of the analysis and reason for dropout |
| Assay Method   1. Sample handling   State the method of storage   1. Assay method   State the type of assay method used to measure GDF15   1. Manufacturer   State the name of company which makes the assay for GDF15   1. Cutpoint   State methods used to determine cutoff |
| Outcomes   1. Confounders   State the conventional risk factors (e.g. age, gender, deep of tumor, lymph node metastasis) or other biomarkers relating with the disease   1. Clinical endpoint   State the clinical endpoint |
| 1. Validation   State the outcome events checked by independent source (e.g. medical records, outpatient visits, by letter and by telephone) |
| Analysis   1. Univariate estimate   Report the effect of GDF15 on outcome   1. Multivariate estimate   Adjusted for confound factors list above.   1. Missing value   State the number of patients with missing value for GDF15 or confounders and how to deal with it. |

**Checklist** The PRISMA checklist

| **Section/topic** | **#** | **Checklist item** | **Reported on page #** |
| --- | --- | --- | --- |
| **TITLE** | | |  |
| Title | 1 | Identify the report as a systematic review, meta-analysis, or both. | 1 |
| **ABSTRACT** | | |  |
| Structured summary | 2 | Provide a structured summary including, as applicable: background; objectives; data sources; study eligibility criteria, participants, and interventions; study appraisal and synthesis methods; results; limitations; conclusions and implications of key findings; systematic review registration number. | 2 |
| **INTRODUCTION** | | |  |
| Rationale | 3 | Describe the rationale for the review in the context of what is already known. | 3 |
| Objectives | 4 | Provide an explicit statement of questions being addressed with reference to participants, interventions, comparisons, outcomes, and study design (PICOS). | 4 |
| **METHODS** | | |  |
| Protocol and registration | 5 | Indicate if a review protocol exists, if and where it can be accessed (e.g., Web address), and, if available, provide registration information including registration number. | 5 |
| Eligibility criteria | 6 | Specify study characteristics (e.g., PICOS, length of follow-up) and report characteristics (e.g., years considered, language, publication status) used as criteria for eligibility, giving rationale. | Supplementary  file |
| Information sources | 7 | Describe all information sources (e.g., databases with dates of coverage, contact with study authors to identify additional studies) in the search and date last searched. | 6 |
| Search | 8 | Present full electronic search strategy for at least one database, including any limits used, such that it could be repeated. | 6 |
| Study selection | 9 | State the process for selecting studies (i.e., screening, eligibility, included in systematic review, and, if applicable, included in the meta-analysis). | 6 |
| Data collection process | 10 | Describe method of data extraction from reports (e.g., piloted forms, independently, in duplicate) and any processes for obtaining and confirming data from investigators. | 6 |
| Data items | 11 | List and define all variables for which data were sought (e.g., PICOS, funding sources) and any assumptions and simplifications made. | Nr |
| Risk of bias in individual studies | 12 | Describe methods used for assessing risk of bias of individual studies (including specification of whether this was done at the study or outcome level), and how this information is to be used in any data synthesis. | 5-6 |
| Summary measures | 13 | State the principal summary measures (e.g., risk ratio, difference in means). | 5-6 |
| Synthesis of results | 14 | Describe the methods of handling data and combining results of studies, if done, including measures of consistency (e.g., I^2^) for each meta-analysis. | 5-6 |

Page 1 of 2

| **Section/topic** | **#** | **Checklist item** | **Reported on page #** |
| --- | --- | --- | --- |
| Risk of bias across studies | 15 | Specify any assessment of risk of bias that may affect the cumulative evidence (e.g., publication bias, selective reporting within studies). | 5-6 |
| Additional analyses | 16 | Describe methods of additional analyses (e.g., sensitivity or subgroup analyses, meta-regression), if done, indicating which were pre-specified. | 5-6 |
| **RESULTS** | | |  |
| Study selection | 17 | Give numbers of studies screened, assessed for eligibility, and included in the review, with reasons for exclusions at each stage, ideally with a flow diagram. | 8 |
| Study characteristics | 18 | For each study, present characteristics for which data were extracted (e.g., study size, PICOS, follow-up period) and provide the citations. | Supplementary  file |
| Risk of bias within studies | 19 | Present data on risk of bias of each study and, if available, any outcome level assessment (see item 12). | 8-10 |
| Results of individual studies | 20 | For all outcomes considered (benefits or harms), present, for each study: (a) simple summary data for each intervention group (b) effect estimates and confidence intervals, ideally with a forest plot. | 8-10 |
| Synthesis of results | 21 | Present results of each meta-analysis done, including confidence intervals and measures of consistency. | 8-10 |
| Risk of bias across studies | 22 | Present results of any assessment of risk of bias across studies (see Item 15). | 8-10 |
| Additional analysis | 23 | Give results of additional analyses, if done (e.g., sensitivity or subgroup analyses, meta-regression [see Item 16]). | 8-10 |
| **DISCUSSION** | | |  |
| Summary of evidence | 24 | Summarize the main findings including the strength of evidence for each main outcome; consider their relevance to key groups (e.g., healthcare providers, users, and policy makers). | 10-12 |
| Limitations | 25 | Discuss limitations at study and outcome level (e.g., risk of bias), and at review-level (e.g., incomplete retrieval of identified research, reporting bias). | 12 |
| Conclusions | 26 | Provide a general interpretation of the results in the context of other evidence, and implications for future research. | 13 |
| **FUNDING** | | |  |
| Funding | 27 | Describe sources of funding for the systematic review and other support (e.g., supply of data); role of funders for the systematic review. | 13 |

*From:*  Moher D, Liberati A, Tetzlaff J, Altman DG, The PRISMA Group (2009). Preferred Reporting Items for Systematic Reviews and Meta-Analyses: The PRISMA Statement. PLoS Med 6(6): e1000097. doi:10.1371/journal.pmed1000097
